# Implant Dentistry: New Materials and Technologies

**DOI:** 10.1155/2019/1465368

**Published:** 2019-04-17

**Authors:** Luigi Canullo, Carlo Mangano, Henriette Lerner

**Affiliations:** ^1^Private Practice, Rome, Italy; ^2^University of Valencia, Valencia, Spain; ^3^Digital Dentistry, Dept. of Dental Sciences, University Vita Salute San Raffaele, Milan, Italy; ^4^Head of Digital Dentistry Unit Research, IRCCS San Raffaele Hospital, Milan, Italy; ^5^Faculty of Dentistry, University of Granada, Spain; ^6^Universidad Catolica, Murcia, Spain; ^7^Private Practice, Gravedona (CO), Italy; ^8^HL Dentclinic and Academy, Baden-Baden, Germany; ^9^Academic Teaching and Research Institution of Johann Wolfgang Goethe-University, Frankfurt am Main, Germany

Implant dentistry is living a revolution of materials and technologies aiming at a higher predictability, quantification, less invasivity, and higher esthetic expectations.

A new fresh sequence of researches is renewing the concepts and predictability of bone grafting implant designs, digital dentistry.

Predictable parameter of treatments of peri-implantitis is creating new perspectives.

Bone grafting philosophy takes a new direction. Researches are mirroring the biological aspects focusing on biological integration and the necessity of lack of foreign body reaction.

New materials and classifications of lateral and vertical grafting are on the way, aiming at a higher predictability and success rate. Collagen matrices have shown capacity of increasing the peri-implantary soft tissue quality and quantity.

Accuracy and precision are the new parameters in implant placement. We take advantage of quantified data about precision and accuracy of digital impressions. Next accuracy and precision in all the new technologies of guided implant placement are taken “under the loupe”: dynamic navigation, planning systems, printing technologies, and surgical guides.

Implant Dentistry never sleeps.

The special issue “Implant Dentistry: New Materials and Technologies” introduces with freshness and professionality the scientific reliability of a new era in implant dentistry.

